# Influencing Factors on the Success of Mobile Learning: A Systematic Review and Meta-Analysis

**DOI:** 10.17533/udea.iee.v42n3e09

**Published:** 2024-10-21

**Authors:** Abdollah Mehrfar, Zahra Zolfaghari, Arash Bordbar, Zahra Mohabbat

**Affiliations:** 1 Student research committee of Shiraz University of Medical Sciences, Shiraz, Iran https://orcid.org/0000-0001-5115-1069 Shiraz University Student research committee Shiraz University of Medical Sciences Shiraz Iran; 2 E-Learning in Medical Sciences Department, Virtual Faculty of Shiraz University of Medical Sciences, Shiraz, Iran. Email: matin_amehrfar2014@yahoo.com https://orcid.org/0000-0002-2986-4262 Shiraz University Learning in Medical Sciences Department Virtual Faculty Shiraz University of Medical Sciences Shiraz Iran matin_amehrfar2014@yahoo.com; 3 Student research committee of Shiraz University of Medical Sciences, Shiraz, Iran https://orcid.org/0000-0002-5464-2426 Shiraz University Student research committee Shiraz University of Medical Sciences Shiraz Iran; 4 E-Learning in Medical Sciences Department, Virtual Faculty of Shiraz University of Medical Sciences, Shiraz, Iran. Email: zzolfagharimedical@gmail.com Corresponding author. https://orcid.org/0009-0000-1647-302X Shiraz University Learning in Medical Sciences Department Virtual Faculty Shiraz University of Medical Sciences Shiraz Iran zzolfagharimedical@gmail.com; 5 FASA University of Medical Science, Fasa, Iran. Email: Arash.bordbar@gmail.com Fasa University FASA University of Medical Science Fasa Iran Arash.bordbar@gmail.com; 6 Healthcare Management, School of Management and Medical Informatics, Shiraz University of Medical Sciences, Shiraz, Iran. Email: Mhbtzahra@gmail.com Shiraz University Iran

**Keywords:** academic success, health education, mobile applications, learning, éxito académico, educación en salud, aplicaciones móviles, aprendizaje, sucesso acadêmico, educação em saúde, aplicativos móveis, aprendizagem

## Abstract

**Objective.:**

To study the geographical regions, success factors, and types of mobile device features that could result in educational success and early take-up.

**Methods.:**

This systematic review and meta-analysis searched PubMed, CINAHL, EMBASE, PsycINFO and ProQuest databases between 2010 and November 2022. The keywords were m-learning features, practical experiences, and influencing. Comprehensive Meta-Analysis software was used to analyze and combine data.

**Results.:**

48 articles were reviewed in this study. Compatibility and user-friendliness of mobile phones were mentioned as key factors influencing the use of mobile devices in learning. Also, the key role of users’ perspectives, attitudes, and skills as determinant factors of applying mobile technology in the learning process was revealed, which confirms its significant role in the success of m-Learning. Other influencing factors were tools readiness, the availability of appropriate resources, motivation of learners and their active engagement, support, and learning styles which considerably could play a key role in improving the quality of m-learning. Applying different strategies including collaboration, effective interaction, reflection, or inquiry-based learning can be beneficial in improving the success rate of m-learning. The final factor was technical competence which showed a significantly negative correlation with m-learning success according to learners’ perspective. The meta-analysis indicated that most studies on mobile learning were conducted between 2015 and 2021, primarily utilizing quantitative methodologies. These studies focused on young adults and were carried out in various countries, including the United States, Spain, Taiwan, Saudi Arabia, the United Kingdom, Turkey, China, Australia, Italy, Sri Lanka, Malaysia, Oman, Austria, South Africa, Egypt, India, Portugal, Jordan, South Korea, Iran, Finland, Brazil, and Israel. A meta-analysis identified 23 countries, with the United States having the highest number of studies on mobile learning success factors. Key determinants reported were learning approach and learners’ perception, with estimates of 0.68 (95% CI 0.06-0.98) and 0.44 (95% CI 0.33-0.56), respectively. In contrast, Jordan and Iran had the lowest number of studies, with learning approach being the main contributing success factor from the learners’ perspective, estimated at 0.736 (95% CI 0.68-0.78)

**Conclusion.:**

Successful m-learning should include the investigation of trainees’ educational needs and motivation; provision of adequate infrastructure and learning materials; definition of learning objectives and course contents; and coordination of appropriate learning activities in order to ensure continuous progress in learners’ knowledge and awareness on different course topics.

## Resumen

**Objetivo.:**

Estudiar las regiones geográficas, los factores de éxito y los tipos de características de los dispositivos móviles que podrían propiciar el éxito educativo y la adopción temprana de este tipo de aprendizaje.

**Métodos.:**

En esta revisión sistemática y metanálisis se realizaron búsqueda de artículos en las bases de datos PubMed, CINAHL, EMBASE, PsycINFO y ProQuest entre 2010 y noviembre de 2022. Las palabras clave fueron características del m-learning, experiencias prácticas e influencia. Se utilizó el software Comprehensive Meta-Analysis para analizar y combinar los datos.

**Resultados.:**

Se revisaron 48 artículos. La compatibilidad y la facilidad de uso de los teléfonos móviles se mencionaron como factores clave que influyen en el uso de dispositivos móviles en el aprendizaje. También se reveló el papel clave de las perspectivas, actitudes y habilidades de los usuarios como factores determinantes de la aplicación de la tecnología móvil en el proceso de aprendizaje, lo que confirma su importante papel en el éxito del m-Learning. Otros factores influyentes fueron la disposición de las herramientas, la disponibilidad de recursos apropiados, la motivación de los alumnos y su compromiso activo, el apoyo y los estilos de aprendizaje, que considerablemente podrían desempeñar un papel clave en la mejora de la calidad del aprendizaje móvil. La aplicación de diferentes estrategias, como la colaboración, la interacción efectiva, la reflexión o el aprendizaje basado en la investigación, puede ser beneficiosa para mejorar el índice de éxito del m-learning. El último factor fue la competencia técnica, que mostró una correlación significativamente negativa con el éxito del m-learning según la perspectiva de los alumnos. El metaanálisis indicó que la mayoría de los estudios sobre aprendizaje móvil se realizaron entre 2015 y 2021, utilizando principalmente metodologías cuantitativas. Estos estudios se centraron en adultos jóvenes y se llevaron a cabo en diversos países, como Estados Unidos, España, Taiwán, Arabia Saudí, Reino Unido, Turquía, China, Australia, Italia, Sri Lanka, Malasia, Omán, Austria, Sudáfrica, Egipto, India, Portugal, Jordania, Corea del Sur, Irán, Finlandia, Brasil e Israel. Los factores determinantes entre los estudios de 23 países fueron el enfoque del aprendizaje y la percepción de los alumnos, con estimaciones de 0.68 (IC95% 0.06-0.98) y 0.44 (IC95% 0.33-0.56), respectivamente. Por el contrario, Jordania e Irán presentaron el menor número de estudios, siendo el enfoque de aprendizaje el principal factor de éxito desde la perspectiva de los alumnos, con una estimación de 0.73 (IC95% 0.68-0.78).

**Conclusión.:**

El éxito del m-learning debe incluir la determinación de las necesidades educativas y la motivación de los alumnos; la provisión de infraestructuras y materiales de aprendizaje adecuados; la definición de los objetivos de aprendizaje y los contenidos del curso; y la coordinación de las actividades de aprendizaje apropiadas para garantizar un progreso continuo en el conocimiento y la concienciación de los alumnos sobre los diferentes temas del curso.

## Resumo

**Objetivo.:**

Estude as regiões geográficas, os fatores de sucesso e os tipos de recursos dos dispositivos móveis que podem impulsionar o sucesso educacional e a adoção precoce desse tipo de aprendizagem.

**Métodos.:**

Nesta revisão sistemática e meta-análise, foram pesquisados artigos nas bases de dados PubMed, CINAHL, EMBASE, PsycINFO e ProQuest entre 2010 e novembro de 2022. As palavras-chave foram características de m-learning, experiências práticas e influência. Um software abrangente de meta-análise foi usado para analisar e combinar os dados.

**Resultados.:**

Foram revisados 48 artigos. A compatibilidade do telemóvel e a facilidade de utilização foram citadas como fatores-chave que influenciam a utilização de dispositivos móveis na aprendizagem. Foi também revelado o papel fundamental das perspectivas, atitudes e competências dos utilizadores como fatores determinantes na aplicação da tecnologia móvel no processo de aprendizagem, confirmando o seu importante papel no sucesso do m-Learning. Outros fatores influentes foram a disposição das ferramentas, a disponibilidade de recursos adequados, a motivação e o envolvimento activo dos alunos, o apoio e os estilos de aprendizagem, que poderiam desempenhar consideravelmente um papel fundamental na melhoria da qualidade da aprendizagem móvel. A aplicação de diferentes estratégias, como colaboração, interação eficaz, reflexão ou aprendizagem baseada em investigação, pode ser benéfica para melhorar a taxa de sucesso do m-Learning. O último fator foi a competência técnica, que apresentou correlação significativamente negativa com o sucesso do m-Learning na perspectiva dos alunos. A meta-análise indicou que a maioria dos estudos sobre aprendizagem móvel foram realizados entre 2015 e 2021, utilizando principalmente metodologias quantitativas. Estes estudos centraram-se em adultos jovens e foram realizados em vários países, incluindo Estados Unidos, Espanha, Taiwan, Arábia Saudita, Reino Unido, Turquia, China, Austrália, Itália, Sri Lanka, Malásia, Omã, Áustria, África do Sul, Egito, Índia, Portugal, Jordânia, Coreia do Sul, Irão, Finlândia, Brasil e Israel. Os fatores determinantes entre os estudos de 23 países foram a abordagem de aprendizagem e a percepção dos alunos, com estimativas de 0.68 (IC 95% 0.06-0.98) e 0.44 (IC 95% 0.33-0.56), respectivamente. Pelo contrário, a Jordânia e o Irão apresentaram o menor número de estudos, sendo a abordagem de aprendizagem o principal fator de sucesso na perspectiva dos alunos, com uma estimativa de 0.73 (IC 95% 0.68-0.78).

**Conclusão.:**

O sucesso do m-Learning deve incluir a determinação das necessidades educativas e da motivação dos alunos; o fornecimento de infraestruturas e materiais de aprendizagem adequados; a definição dos objetivos de aprendizagem e dos conteúdos do curso; e a coordenação de atividades de aprendizagem adequadas para garantir o progresso contínuo no conhecimento e consciência dos alunos sobre os diferentes temas do curso.

## Introduction

The widely prevalent use of technology in today’s life, the continuous updating of information, and the growing need of people to access information without any time and place restrictions as well as the individualization of education have resulted in an emergence of new approaches such as e-learning and mobile learning.^1^ On the other hand, wireless technologies and portable electronic devices played a key role in the development of such educational methods.^2^ In fact, mobile technology is not only a means to communicate at any time and place, but it is also capable of providing unlimited information in educational fields through the use of developed applications.^3^ Mobile learning or m-Learning is the subset of distance learning which uses mobile technologies including mobile phones, tablets, and laptops in the learning process.^4^ This method brings several advantages for both teachers and trainees including mobility, quick access to information, time efficiency, personalization, diversity, and flexibility.^5^^,^^6^ Moreover, it fosters collaborative learning, and rapid feedback which consequently provides social negotiation space and facilitates effective interaction between students and trainers.^7^ In a study conducted by Fu and Hwang, it was emphasized that collaborative learning through mobile devices has the potential to increase learners' intellectual and meta-cognitive development.^8^ Furthermore, Ozdamli and Cavus highlighted that the core characteristics of mobile learning including individual, collaborative, cooperative, adaptable, and instant transfer of information enable trainees to experience a faithful delight of learning.^9^ In fact, with the arrival of mobile devices and smartphones, learning aided by mobile applications can avoid cognitive exhaustion and synaptic fatigue and consequently, they have the potential to efficiently maintain the mechanisms of cognitive task performance. A similar research conducted in Australia found that students had a significant desire to participate in more cooperative learning activities comprised of mobile technologies.^10^ Furthermore, they revealed a great sense of commitment to a diverse range of digital tools, and distance learning approaches. Accordingly, the literature affirmed that trainees might feel more encouraged when using mobile technologies in learning.^11^^-^^13^

The significant increase in the number of mobile phones per 100 people, from 12.075 in 2000 to 98.622 in 2015 emphasizes that the utilization of mobile devices has become almost worldwide.^14^ Accordingly, it is expected that such evolving technologies are progressively used for educational purposes.^15^ In fact, besides the use of mobile devices in all aspects of people’s lives across the globe, their significant impact on education and learning process which can happen in collaborative, and real life contexts has been emphasized in the literature.^16^^-^^19^ As higher education graduates are expected to have creativity, engage in rational decision making, and solve problems in a systematic way, on-line courses through the use of mobile devices might be more efficient for improving their thinking skills. Despite the significant expansion of this technology in the field of education, there are still definite barriers to adoption of a successful m-Learning platform, particularly in higher learning institutions.^20^To resolve the issue, several studies have been conducted worldwide to determine the success factors of m-learning in higher education. Some research aimed to figure out the effect of demographics on the success of m-learning and some others mentioned the long-term use of this learning method as an important factor for its success and effective impact on students' learning capabilities.^21^^,^^22^ As these research works mainly concentrate on a particular feature of the m-learning program, this study is going to have a more holistic view of the subject and conduct a systematic review and meta-analysis to organize the studies in terms of factors such as geographical regions, success factors, and types of mobile devices which could be important for educational success and early take-up from students.

## Methods

A systematic review was conducted based on the Preferred Reporting Items for Systematic reviews and Meta-Analyses guidelines (PRISMA).^23^ This systematic review follows some of the previous works in this area, including a research developed by Alrasheedi *et al.*^20^ However, in this study we focused on conducting a systematic review of the literature published since the onset of the 2010 decade and organized a meta-analysis in order to present a series of influencing factors on the success of mobile learning in higher education. 

*Systematic review procedure.* To conduct the systematic review, we followed the procedure consisted of three main phases including planning of systematic mapping; conducting the review; and reporting the review. In the first phase, we conducted a comprehensive search to investigate related studies performed in mobile learning and discover the gap of the existing systematic reviews. The research questions which have been formulated in this review aimed to gain adequate information to determine the critical success factors of mobile learning in higher education. The research questions were: Which countries/ or geographic areas have implemented mobile learning? What are the influencing factors on the success of mobile learning implementation? Which types of mobile devices are more important for mobile educational success?

*Databases and search terms.* To find relevant studies, electronic databases that include the majority of articles and conference papers associated with the field of mobile learning in higher education including PubMed (MEDLINE), CINAHL, EMBASE, PsycINFO and ProQuest were reviewed within the period of 2010 to 2022. Since the use of mobile devices in education has been seriously considered since the 20^th^ century, older studies were excluded as their findings might no longer be valid to current mobile learning contexts.^24^^-^^26^ In the phase of conducting the review, the keywords which have been used in the searching process were “m-learning”, “mobile learning”, “applications”, “mobile devices”, “mobile apps”, “mobile collaborative learning”, “collaborative learning”, “cooperative learning”, "ubiquitous learning”, “critical success factors”, “key performance indicators” and “higher education”. We limited the search to articles with full-text access and published in English. In order to retrieve the maximum number of relevant papers, we also reviewed the reference list of included papers. 

**Figure 1:**
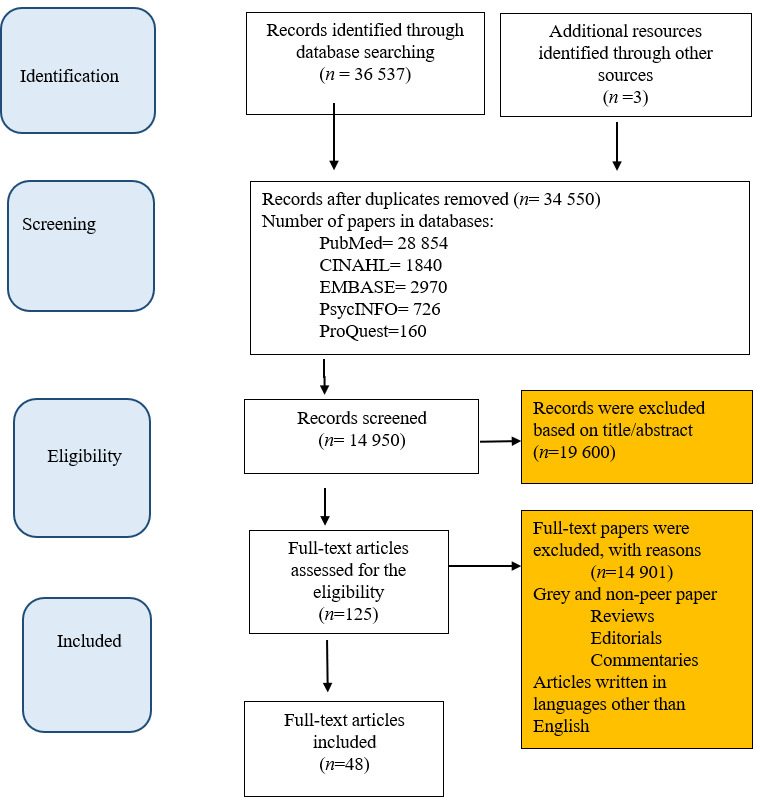
Prisma Diagram

*Inclusion and exclusion criteria.* Then, an initial review of all abstracts was done and followed by an in-depth review of selected articles according to their relevance with study objectives and inclusion criteria including (1) peer-reviewed journal articles, (2) studies containing mobile learning, success factors, and early take-up among students, (3) full-text access, (4) higher education, (5) mobile collaborative learning, and (6) investigation of success factors for m-learning. On the other hand, studies of mobile learning that involved lower educational levels such as school students or kindergarten children were excluded from the review. Furthermore, papers without full-text access or published in languages other than English were not considered for further review. After reviewing the titles and abstracts of searched papers based on the inclusion and exclusion criteria, the full texts of the papers that could not be removed were reviewed. During this process, 19600 papers were excluded only by reading the titles and abstract, and 14901 by reviewing the full text. Figure 1 shows the process of searching and selecting primary papers. 

*Data extraction.* Two independent investigators used a data extraction form including the name of author/ authors, date of publication, research setting, study design, and a brief of study findings. In terms of any disagreement, a third reviewer was asked to resolve the issue. 

*Quality Assessment.* To assess the methodological quality of included studies, two independent researchers used the Newcastle-Ottawa Scale (NOS). The scale contains eight questions in three main sections including exposure/ outcome ascertainment, selection of study groups, and their comparability. The number of possible answers per question ranges between two and five. A study with score between 7 to 9, was considered as high quality, 4 to 6 as high risk, and 0 to 3 as very high risk of bias. To achieve consensus in case of any discrepancy, we consulted with a third party.^27^

*Statistical Analysis.* After reviewing the details of each primary article, content analysis was conducted and the studies were coded for the classifications including: author/ authors’ name, year of publication, study objective, methodology, and findings. To determine heterogeneity based on different learners’ geographical regions, success factors, and types of mobile devices subgroup analysis was used. Meta-analysis was performed using Comprehensive Meta-Analysis software.

## Results

According to the search procedure, a total number of 48 articles were extracted. Figure 1 shows the process of selecting studies included in the meta-analysis during the literature review based on Preferred Reporting Items for Systematic Reviews and Meta-analysis (PRISMA) guideline. 

Study findings revealed that the majority of studies on mobile learning was conducted between the years 2015 and 2021, used quantitative methodology, carried on among young adults and in countries including United States, Spain, Taiwan, Saudi Arabia, United Kingdom, Turkey, China, Australia, Italy, Sri Lanka, Malaysia, Oman, Austria, South Africa, Egypt, India, Portugal, Jordan, South Korea, Iran, Finland, Brazil and Israel. 

*Meta-analysis based on Countries.* Based on meta-analysis, 23 countries were identified. Among the countries, United States incorporated the maximum number of research on mobile learning success factors reporting learning approach and learners’ perception as key determinants estimated at 0.687 (95% CI, 0.063-0.986) and 0.447 (95% CI, 0.337-0.562) respectively. Whereas, Jordan and Iran were reported to have the lowest number of studies on mobile learning mentioning learning approach as the main contributing success factor from the learners’ perspective estimated at 0.736 (95% CI, 0.685-0.781) (Table 1). The summary of selected studies is depicted in Table 2.

**Table 1:** Meta-analysis based on Countries

Sub-groups		Effect size and 95% interval			Test of null (2-Tail)	
		Point estimate	Lower limit	Upper limit	Z-value	P-value
United states	Device features	0.234	0.185	0.291	-0.876	<0.0001
	Learners’ perception	0.447	0.337	0.562	-0.904	0.366
	Learning approach	0.687	0.063	0.986	0.443	0.658
	Pedagogical benefits	0.356	0.299	0.418	-1.422	<0.0001
China	Device features	0.345	0.179	0.366	-0.425	0.249
	Learners’ perception	0.425	0.277	0.455	−2.027	<0.0001
	Learning approach	0.435	0.322	0.574	−0.602	0.167
	Pedagogical benefits	0.267	0.162	0.472	0.522	0.114
United Kingdom	Device features	0.567	0.542	0.611	2.146	0.005
	Learners’ perception	0.695	0.215	0.561	-2.148	0.005
	Learning approach	0.588	0.542	0.633	3.709	<0.0001
	Pedagogical benefits	0.366	0.344	0.741	−4.385	<0.0001
Taiwan	Device features	0.227	0.149	0.312	−2.047	0.586
	Learners’ perception	0.315	0.287	0.466	−2.122	0.214
	Learning approach	0.308	0.186	0.355	−2.077	0.645
	Pedagogical benefits	0.245	0.118	0.356	−1.042	0.127
Spain	Device features	0.596	0.512	0.651	2.488	0.005
	Learners’ perception	0.588	0.506	0.614	-2.312	0.005
	Learning approach	0.658	0.872	8.125	0.000	0.637
	Pedagogical benefits	0.214	0.551	0.316	−2.953	<0.0001
Malaysia	Device features	0.060	0.066	0.072	-13.833	<0.0001
	Learners’ perception	0.471	0.162	0.221	-10.833	<0.0001
	Learning approach	0.367	0.238	0.483	−8.042	<0.0001
	Pedagogical benefits	0.042	0.077	0.082	-18.338	<0.0001
South Africa	Device features	0.014	0.010	0.025	-12.072	<0.0001
	Learners’ perception	0.074	0.015	0.298	−2.970	0.003
	Learning approach	0.116	0.132	0.268	−6.180	<0.0001
	Pedagogical benefits	0.189	0.142	0.293	−7.166	<0.0001
Australia	Device features	0.518	0.442	0.678	3.104	<0.0001
	Learners’ perception	0.541	0.425	0.663	-3.907	<0.0001
	Learning approach	0.598	0.514	0.733	3.908	<0.0001
	Pedagogical benefits	0.314	0.542	0.633	3.709	<0.0001
India	Device features	0.233	0.115	0.298	-1.174	<0.0001
	Learners’ perception	0.376	0.198	0.211	-3.978	<0.0001
	Learning approach	0.332	0.157	0.322	-3.786	<0.0001
	Pedagogical benefits	0.188	0.142	0.353	1.907	<0.0001
Sri Lanka	Device features	0.229	0.132	0.365	2.385	<0.0001
	Learners’ perception	0.278	0.132	0.427	-2.562	<0.0001
	Learning approach	0.423	0.127	0.356	2.146	<0.0001
	Pedagogical benefits	0.179	0.112	0.342	2.586	<0.0001
Egypt	Device features	0.122	0.137	0.256	-1.768	<0.0001
	Learners’ perception	0.229	0.244	0.457	-3.667	<0.0001
	Learning approach	0.342	0.213	0.357	-2.876	<0.0001
	Pedagogical benefits	0.150	0.149	0.157	-1.166	<0.0001
Finland	Device features	0.168	0.121	0.215	1.872	0.005
	Learners’ perception	0.369	0.321	0.626	2.148	0.005
	Learning approach	0.599	0.512	0.718	3.449	0.005
	Pedagogical benefits	0.165	0.182	0.514	2.418	0.005
Portugal	Device features	0.355	0.285	0.577	2.336	<0.0001
	Learners’ perception	0.368	0.225	0.561	3.423	<0.0001
	Learning approach	0.563	0.285	0.671	5.423	<0.0001
	Pedagogical benefits	0.129	0.062	0.250	-4.639	<0.0001
Jordan	Device features	0.029	0.062	0.250	-0.937	<0.0001
	Learners’ perception	0.043	0.062	0.250	-2.639	<0.0001
	Learning approach	0.037	0.066	0.228	-3.936	<0.0001
	Pedagogical benefits	0.029	0.042	0.152	-1.369	<0.0001
Saudi Arabia	Device features	0.166	0.152	0.206	-15.692	<0.0001
	Learners’ perception	0.311	0.052	0.601	−0.458	0.510
	Learning approach	0.366	0.335	0.659	−1.604	0.004
	Pedagogical benefits	0.256	0.157	0.266	-9.962	<0.0001
South Korea	Device features	0.283	0.149	0.417	-0.485	<0.0001
	Learners’ perception	0.391	0.172	0.489	−2.920	0.176
	Learning approach	0.371	0.271	0.482	−2.670	0.011
	Pedagogical benefits	0.177	0.292	0.386	−1.495	0.014
Iran	Device features	0.036	0.070	0.408	-4.289	<0.0001
	Learners’ perception	0.055	0.064	0.309	1.853	<0.0001
	Learning approach	0.069	0.032	0.696	3.385	<0.0001
	Pedagogical benefits	0.047	0.057	0.286	2.53	<0.0001
Turkey	Device features	0.192	0.132	0.298	−4.250	<0.0001
	Learners’ perception	0.286	0.148	0.384	−3.217	<0.0001
	Learning approach	0.426	0.359	0.495	-2.103	0.035
	Pedagogical benefits	0.014	0.025	0.299	−2.770	0.003
Austria	Device features	0.127	0.231	0.325	−2.144	<0.0001
	Learners’ perception	0.268	0.183	0.483	−2.712	<0.0001
	Learning approach	0.456	0.389	0.465	-2.103	0.035
	Pedagogical benefits	0.018	0.028	0.287	−2.076	0.003
Israel	Device features	0.0892	0.0134	0.287	−3.502	<0.0001
	Learners’ perception	0.216	0.148	0.384	−3.127	<0.0001
	Learning approach	0.475	0.359	0.432	-2.301	0.035
	Pedagogical benefits	0.012	0.021	0.280	−1.707	0.003
Oman	Device features	0.026	0.042	0.458	-3.208	<0.0001
	Learners’ perception	0.036	0.059	0.326	1.764	<0.0001
	Learning approach	0.072	0.022	0.296	3.385	<0.0001
	Pedagogical benefits	0.037	0.047	0.256	2.53	<0.0001
Italy	Device features	0.237	0.136	0.312	-0.485	<0.0001
	Learners’ perception	0.318	0.152	0.397	−2.920	0.001
	Learning approach	0.363	0.251	0.288	−2.670	<0.0001
	Pedagogical benefits	0.142	0.266	0.393	−1.495	<0.0001
Brazil	Device features	0.016	0.080	0.302	-2.892	<0.0001
	Learners’ perception	0.155	0.164	0.309	2.538	<0.0001
	Learning approach	0.069	0.032	0.696	3.552	<0.0001
Pedagogical benefits	0.022	0.036	0.268	2.437	<0.0001

**Table 2:** The summary of selected studies from the year 2010 to 2022

Althunibat (2015). Country: Jordan. Study design: survery. Findings: The proposed model of m-learning is comprehensive to study in the institutions of higher education.
Briz-Ponce et al. (2017). Country: Portugal. Study design: survery. Findings: Technology Acceptance Model was affirmed to be applied within the context of Innovation in Education.
Karimi et al. (2015). Country: UK. Study design: Case-control. Findings: The role of learners’ characteristics in m-learning adoption was approved and the importance of distinguishing between various types of m- learning was highlighted.
Koc et al. (2016). Country: Turkey. Study design: Structural equation modeling. Findings: Strong exogenous role of context and a positive strong relationship among perceived ease of use, perceived usefulness and trust to intentions to use were confirmed in the study.
Oberer and Erkollar. (2011). Country: Austria. Study design: Survey. Findings: The advantages of mobile learning modules in higher education were approved
Ooi et al. (2018). Country: Malaysia. Study design: Structural equation modeling. Findings: Perceived mobility and social presence affected satisfaction indirectly through mobile usefulness and sense of belonging
Parsazadeh et al. (2018). Country: Iran. Study design: Case-control. Findings: The applicability of the device was significantly effective in improving students’ online information evaluation skills.
Shorfuzzaman and Alhussein. (2016). Country: Saudi Arabia. Study design: Empirical study. Findings: A model was proposed to investigate learners’ readiness to adopt M-learning.
So S. (2016). Country: China. Study design: Case-control. Findings: The use of a MIM tool (WhatsApp) was affirmed to support teaching and learning objectives in higher education
Cho et al. (2017). Country: South Korea. Study design: Case-control. Findings: Self-regulated learning is important in cultivating positive community of inquiry
Jones et al. (2013). Country: UK. Study design: Case study. Findings: nQuire could support learners’ inquiries in an informal context without teachers presence
Molinillo et al. (2018). Country: Spain. Study design: Survey. Findings: Flow, active learning and perceived benefits of m-learning can influence on trainees’ attitude.
Reychav and Wu. (2016). Country: Israel. Study design: Survey. Findings: Educators need to balance the interface design of mobile training systems and different complexity levels of cognitive tasks in various training domains, in order to achieve the desired training outcomes.
Sánchez-Prieto et al. (2017). Country: Spain. Study design: Structural equation modeling. Findings: Trainees perceived usefulness and behavioral intention, perceived ease of use and perceived usefulness, and self-efficacy of m-learning.
Al-Otaibi et al. (2016). Country: Saudi Arabia. Study design: Case study. Findings: High usability rates and generally positive attitudes toward using the mobile lab system were affirmed.
Cheon et al. (2012). Country: USA. Study design: Survey. Findings: Perceived behavioral control was the main determinant of m-learning adoption.
Gikas and Grant. (2013). Country: USA. Study design: Qualitative research. Findings: Mobile devices and the use of social media create opportunities for interaction, collaboration, and students’ engagement in content creation and communication using social media and Web tools.
Lin and Lin. (2015). Country: Taiwan. Study design: Case-control. Findings: M-learning is helpful to students in improving learning performance and reducing cognitive loads.
Witt et al. (2016). Country: USA. Study design: Survey. Findings: M-learning was useful for students allowing them to access information throughout undergraduate medical education.
Ekanayake and Wishart. (2015). Country: Sri Lanka. Study design: Qualitative study. Findings: M-learning provided teachers with an opportunity for planning and reviewing workshops, using the technology in science teaching and learning, and in sharing knowledge and skills.
Christensen and Knezek. (2018). Country: USA. Study design: Survey. Findings: Educators who are higher in technology integration agree on the usefulness of m-learning, and prefer online or blended learning.
Lackovic et al. (2017). Country: UK. Study design: Qualitative study. Findings: Students perceived Twitter as an employability tool and a tool for transferring knowledge.
Seta et al. (2014). Country: Italy. Study design: Review. Findings: The future of m-learning can be understood as a 360-degree vision that takes into account a range of pedagogical, managerial, political, and ethical issues.
Chang et al. (2016). Country: Taiwan. Study design: Case-control. Findings: Students who participated in the m-learning program showed a significantly higher level of motivation, confidence, and satisfaction.
Chuang Y-T. (2015). Country: Taiwan. Study design: Survey. Findings: The Smartphone-Supported Collaborative Learning System could support collaborative learning.
Lan et al. (2012). Country: Taiwan. Study design: Case-control. Findings: Students who used mobile devices in learning could engage more in reflective thinking, share more information, and facilitate social knowledge construction.
Masters et al. (2016). Country: Oman. Study design: Review. Findings: M-learning can help medical teachers benefit from technological advances at all levels of medical education and improve patient healthcare.
Pimmer et al. (2014). Country: South Africa. Study design: Case study. Findings: Mobile phones, and the convergence of mobile phones and social media, can change learning environments in a constructive way.
Bellina and Missoni (2011). Country: Italy. Study design: Qualitative study. Findings: The possibility to share images on the mobile phone and share information in group discussions proved the usefulness of educational mobile tools.
Gedik et al. (2012). Country: Turkey. Study design: Survey. Findings: The function of mobile instruction was critical in pedagogical aspects; the use of motivational design was also helpful in delivering educational content.
O’Bannon and Thomas (2015). Country: USA. Study design: Survey. Findings: Access to the Internet, clicker capabilities, and the use of educational apps were the most valuable aspects of m-learning. However, disruptions, cyberbullying, and accessing inappropriate content were significant barriers to the use of mobile phones in the classroom.
Al-Emran (2016). Country: Oman. Study design: Not specified. Findings: Significant differences were found among students’ attitudes towards m-learning based on smartphone ownership, country, and age.
Domingo and Badia Garganté (2016). Country: Spain. Study design: Survey. Findings: M-learning helps teachers leverage the combination of mobile technology and apps to improve certain aspects of learning practice.
Melero et al. (2015). Country: Spain. Study design: Survey. Findings: Using mobile phones for learning has a significant positive impact on educational performance.
Leinonen (2014). Country: Finland. Study design: Survey. Findings: There is potential for fostering reflective practices in classroom learning through the use of apps for audio-visual recordings.
Lam and Duan (2012). Country: European countries. Study design: Design research. Findings: There is potential for improving reflective practices in classroom learning through the use of apps for audio-visual recordings.
Scott (2017). Country: Australia. Study design: Mixed-method. Findings: For many students and physicians, the advantages of using mobile devices for learning outweighed the possible risks.
Kuznekoff et al. (2015). Country: Not specified. Study design: Experimental study. Findings: Unrelated messages to class content negatively impacted learning, while related messages did not have a significant negative impact.
Felisoni and Godoi (2018). Country: Brazil. Study design: Survey. Findings: M-learning can be useful for educators and other academic stakeholders interested in using technology to promote the educational performance of trainees.
Jarrahi et al. (2017). Country: USA. Study design: Survey. Findings: The diversity of information and communication technologies affects individuals’ preferences and contextual factors.
Sobaih et al. (2016). Country: Egypt. Study design: Survey. Findings: Social media can be used as an innovative and effective tool for teaching and learning.
Nayak J.K. (2018). Country: India. Study design: Survey. Findings: Female students were less affected by smartphone addiction compared to male students, who experienced neglect of work, anxiety, and loss of control.
Mu and Paparas (2015). Country: UK. Study design: Survey. Findings: Kahoot integrated the advantages of clickers and mobile technology for economics teaching.
Frank and Kapila (2017). Country: USA. Study design: Survey. Findings: Students who used mixed-reality learning environments showed improvement in their knowledge of dynamic systems and control concepts.
Dolawatta et al. (2020). Country: Sri Lanka. Study design: Survey. Findings: The most significant influential factor in the success of m-learning was screen zooming.
Alhumaid et al. (2021). Country: Saudi Arabia. Study design: Structural equation modeling. Findings: Mobile learning in education, amid the coronavirus pandemic, yielded potential outcomes for teaching and learning.
Qashou A. (2021). Country: Not specified. Study design: Not specified. Findings: Perceived usefulness and attitude significantly influenced m-learning adoption intention, while perceived usefulness, perceived ease of use, and perceived self-efficacy significantly affected attitudes toward using m-learning.
Al-Rahmi et al. (2021). Country: Malaysia. Study design: Survey. Findings: The study validated a technology acceptance model, demonstrating that the predicted model effectively predicts students’ attitudes towards using m-learning.

*Influencing factors on mobile learning.* Thirty-three of included research mentioned compatibility and user friendly of mobile phones as key factors influencing the use of mobile devices in learning.^28^^-^^36^ Literature review also revealed the key role of users’ perspectives, attitudes and skills as determinant factors of applying mobile technology in the learning process, which confirms its significant role in the success of m-Learning.^(29,30, 37-41)^ Other influencing factors were regarded as tools readiness, the availability of appropriate resources, motivation of learners and their active engagement, support and learning styles which considerably could play a key role in improving the quality of mobile learning.^29^^,^^42^^-^^49^In fact, applying different strategies including collaboration, effective interaction, reflection, or inquiry-based learning can be beneficial in improving the success rate of m-learning.^36^^,^^50^^-^^54^ These features can be integrated in educational courses through the use of a variety of mobile applications. For instance, trainees can benefit from messenger application to share a particular content either in the form of a text, image or video and consequently make their classmates discuss about the shared contents.^54^^-^^59^ As it is shown in Table 3, both educational content and user friendly design had positive correlations with m-learning success and were mentioned as significant factors by trainees to choose m-learning approach as an effective learning strategy. Ownership which mainly deals with flexibility to use m-learning anytime, anyplace, the possibility to use m-learning platform to connect with other educators, and learners’ perception were also regarded as other contributing factors for the success of m-learning approach. The last factor was technical competence which showed a significantly negative correlation with m-learning success according to learners’ perspective. This means that students in selected studies believed that they already have a quite appropriate technical capability which provides them an opportunity to use m-Learning platform.

**Table 3:** Meta-analysis of Success Factors for m-learning

Success factor categories	Related factors	Effect size and 95% Interval			Test of null (2-Tail)	
		Point estimate	Lower limit	Upper limit	Z-value	P-value
Device features	Compatibility	0.278	0.164	0.372	-3.461	<0.0001
	Functionality and readiness	0.266	0.133	0.469	-7.070	<0.0001
	Availability	0.258	0.158	0.344	-4.095	<0.0001
Learners’ perspective	Self-control	0.232	0.156	0.447	-5.092	0.01
	Flexibility	0.189	0.102	0.254	-2.156	0.01
	Life-long learning	0.065	0.981	0.227	-0.0936	0.01
Pedagogical benefits	Collaborative learning	0.264	0.152	0.414	-3.643	0.01
	Integrative learning	0.225	0.113	0.365	-2.675	<0.0001
	Interactive learning	0.235	0.013	0.129	-1.905	<0.0001
	Learning in context	0.208	0.087	0.356	-3.190	<0.0001
	Problem-based learning	0.0745	0.162	0.374	-3.449	<0.0001
Learning approach	User friendly	0.276	0.142	0.254	-7.500	<0.0001
	Assimilation with curriculum	0.189	0.227	0.284	-4.439	0.012
	Technical competence	0.0298	0.179	0.310	-3.782	<0.0001
	User feed back	0.0542	0.166	0.347	-2.908	<0.0001
Learning community development	0.088	0.214	0.592	-3.763	<0.0001

As data shows in the table, compatibility of the device and user-friendly of the learning approach had substantial effects on the success of m-Learning platform based on the trainees’ experience. Furthermore, as factors including availability of resources, positive attitude of learners toward the learning approach and pedagogical benefits revealed a correspondingly high point estimates, it was proved that each of the mentioned factors had significant impacts on students’ experience with the m-Learning platform.

*Mobile devices.* Regarding mobile learning tools and technologies, most of the studies mentioned mobile phones and tablets as principal component of learning which accordingly allow students to access different sources of information from anywhere, they exist ^(35, 57, 60)^. Literature affirmed that the functionality of mobile devices such as providing social media, images, videos, massages, and virtual learning can help the learning process develop in an effective manner.^(32,33,45, 53,61-68)^ As shown in Table 4, mobile phones, tablets, personal digital assistants (PDAs) and the iPod touch revealed the most compatibility with the needs and desires of learners. In fact, these means of communication have become an integral part of human life, and easily facilitate provide users with appropriate access to the required information in the shortest possible time.^57^ Therefore, these mobile technologies not only make learning possible at any time and place, but also facilitate easy access to some important features such as taking photos and videos, sending SMS, and sharing information through social medias or benefiting from virtual learning environments.^58^^-^^62^^,^^69^^-^^71^

**Table 4:** Meta-analysis based on type of device

Sub-groups	Effect size and 95% interval			Test of null (2-Tail)	
	Point estimate	Lower limit	Upper limit	Z-value	P-value
Mobile phone	0.633	0.412	0.6667	1.082	0.017
Tablet	0.509	0.372	0.635	8.011	<0.0001
Personal digital assistant	0.242	0.146	0.269	-3.988	<0.0001
iPod touch	0.187	0.106	0.215	-2.879	<0.0001

*Meta-regression for Quality Assessment.* In case of quality assessment, more than half of the included studies had high quality, while 17 studies were of medium quality and the rest were of low quality (Table 5).

**Table 5:** Meta-analysis based on quality of studies

Sub-groups		Effect size and 95% interval			Test of null (2-Tail)	
	Number Studies	Point estimate	Lower limit	Upper limit	Z-value	P-value
High	28	0.268	0.176	0.352	-2.701	<0.0001
Low	8	0.159	0.115	0.304	-3.266	<0.0001
Medium	17	0.234	0.198	0.339	-4.627	<0.0001

**Figure 2:**
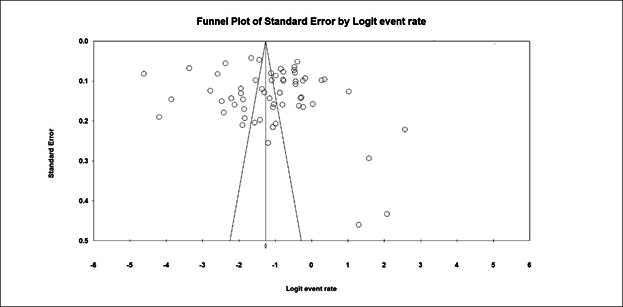
The results of Egger’s statistical test

*Publication bias.*The results of Egger’s statistical test showed the P-value (2-tailed) of 0.48, confirming no publication bias in the study (Figure 2).

## Discussion

In this article, a systematic review and meta-analysis was done to provide a comprehensive review of the existing literature in the field of mobile learning in higher education. Our study identified 48 research conducted in 23 countries worldwide. As findings revealed, the number of studies in the years 2019 to 2022 exceeded the number of papers published in previous years. The reason might be due to the Covid-19 pandemic which led to a significant expansion of distance education and electronic learning after the school and university closures, household quarantine, and social distancing policies. During the pandemic, higher education institutions were forbidden to continue traditional teaching activities and were forced to provide their educational programs through online platforms.^(72, 73)^ On the other hand, today almost all people have access to smart phones and are able to use many programs and features of these devices for various reasons including work, entertainment, leisure and also knowledge acquisition.^74^ Furthermore, our review found that the number of studies involving young adults was higher compared to those studying middle-aged and older adults. This might be due to the fact that young adults have the knowledge and skills of using mobile devices and are frequent users of this technology for various purposes such as entertainment, work and education.^75^ Moreover, due to the widespread closure of universities amid COVID-19 pandemic, the use of mobile phones for educational purposes increased dramatically among college students who are generally in the young age group.^76^

In our review, 16 factors were identified to be crucial for the success of m-Learning. One of the most important factors was compatibility and user friendly, followed by tool readiness, availability of appropriate resources, motivation of learners, support and learning styles, and instructional design. This finding highlights the need for providing an accessible m-Learning platform by academic institutions to ensure the flexibility of learning anytime, and anyplace as well as connecting with other educators even from remote distances. Regarding internet access, education administrators especially in developing countries need to manage courses effectively and consider the necessary infrastructure such as Wi-fi, internet connection for applying mobile devices in their institutes.^77^^,^^78^ Similar studies also mentioned technology-related problems as main barriers for effective mobile learning.^77^^-^^82^ Regarding this category of problems, literature emphasized on key problems that might be evolved due to difficulty in Internet connections, inappropriateness of screen size and keyboard, inconveniences caused by accessories, and distractions during learning through mobile devices.^80^^,^^81^^,^^83^^,^^84^

The next imperative factor was blended learning. In this learning approach, instructors pay particular attention to instructional design, which includes trainees’ analysis, objective identification, learning development, and instructional assessment.^85^^-^^93^ In fact, trainers can benefit from online discussions on mobile devices for increasing learning communication and knowledge sharing between learners to improve learning outside traditional classrooms.^87^^,^^88^^,^^92^^-^^94^ To apply effective strategies that motivate trainees to use their electronic devices for learning purposes, instructors should develop appropriate instructional design focusing on the identification of students’ characteristics and learning styles, their educational needs, and motivation; determination of learning objectives and contents; provision of proper infrastructure and materials; coordination of interactive learning activities; and evaluation of learning activities.^95^^-^^106^ Similarly, literature emphasized that trainers should consider learners', and other instructors' attitudes toward mobile learning approach,^29^^,^^30^^,^^44^^,^^107^ their motivation,^28^^,^^35^ or readiness to take up courses and fulfill them in an effective way.^28^^,^^62^ Therefore, evaluating the course content, materials and tools, and study objectives should be mentioned as dominant factors for recognizing an operative approach for delivering content, coordinate learning activities, and conduct following assessments. In the meantime, learners’ characteristics should be considered in defining learning activities and evaluation strategies.^24^ Moreover, due to an increasing pace of technological growth, instructors should create collaborative learning environments for empowering practical skills and mimicking actual work experiences.^39^ Collaborative learning is regarded as the most common learning strategy that teachers apply through online tools utilizing mobile applications, video conferences and web applications in their courses.^33^^,^^34^^,^^39^^,^^48^^,^^49^ Both proper content and user friendly design of the application are important for learners when choosing m-learning approach.^82^^,^^108^

Lack of fundamental skills in using mobile devices, and negative attitude of instructors towards applying mobile devices in education are among other important barriers to mobile learning.^109^ Learners require some knowledge and skills for using applications in mobile devices, and maintain cyber security.^29^^,^^47^^,^^66^ Instructors also need computer skills and some particular techniques to apply mobile devices in traditional classrooms.^37^^,^^48^ When a new learning approach emerges, it brings about new condition, times and geographies to traditional classrooms. As a result, both teachers and students should be prepared to cope with an evolving technology and expand learning opportunities along with interactive learning methods comprised of investigation, discussion, explanation and lecturing.^1^ Overall, it was found that in order to achieve a long-term success in m-learning, considering the mentioned factors is certainly essential. However, assessing the success factors for making the most of the benefits of m-learning by more detailed research into learners’ demographics and regions revealed that middle-aged adults are the main users of mobile devices in sharing information and pursuing educational purposes. The significant role of such characteristics and individual success factors gives an indication to where the resistance to take-up actually occurs. Therefore, in this study we quantified the impact of each success factor in an accurate statistical term, and mentioned it as a relevant basis for designing future m-Learning instructions. 

Study limitations. This study has a number of limitations which might have influenced the research. First, it does not include m-learning in primary or secondary school contexts, and its only covers the features of mobile learning in higher education contexts. Second, the review was limited to studies published in English; therefore, some relevant studies might not have been involved if they did not fit the language criterion. 

Considering the fact that there are still few studies that address the success factors of mobile devices in the area of education, the novelty of current study is that it provides a holistic view of the subject and conduct a systematic review and meta-analysis to organize the studies in terms of factors such as geographical regions, learner characteristics, and instructional design which could be important for educational success and early take-up. 

Conclusion. The use of mobile learning enables the improvement of lifelong learning under any situation in the future. Study findings suggest that a successful mobile learning should include the investigation of trainees’ educational needs and motivation; provision of adequate infrastructure and learning materials; definition of learning objectives and course contents; and coordination of appropriate learning activities in order to ensure a continuous progress in learners’ knowledge and awareness on different course topics. Furthermore, education administrators should guarantee the availability of Internet connection and the appropriateness of mobile applications for enhanced learning activities. Instructors should also evaluate the effectiveness of m-learning approach regarding to different course topics and manage the existing barriers in an effective manner.
